# The Carrot or the Stick? Evaluation of Education and Enforcement as Management Tools for Human-Wildlife Conflicts

**DOI:** 10.1371/journal.pone.0015681

**Published:** 2011-01-12

**Authors:** Sharon Baruch-Mordo, Stewart W. Breck, Kenneth R. Wilson, John Broderick

**Affiliations:** 1 Graduate Degree Program in Ecology, Department of Fish, Wildlife, and Conservation Biology, Colorado State University, Fort Collins, Colorado, United States of America; 2 USDA-WS-National Wildlife Research Center, Fort Collins, Colorado, United States of America; 3 Colorado Division of Wildlife, Fort Collins, Colorado, United States of America; University of Maribor, Slovenia

## Abstract

Evidence-based decision-making is critical for implementing conservation actions, especially for human-wildlife conflicts, which have been increasing worldwide. Conservation practitioners recognize that long-term solutions should include altering human behaviors, and public education and enforcement of wildlife-related laws are two management actions frequently implemented, but with little empirical evidence evaluating their success. We used a system where human-black bear conflicts were common, to experimentally test the efficacy of education and enforcement in altering human behavior to better secure attractants (garbage) from bears. We conducted 3 experiments in Aspen CO, USA to evaluate: 1) on-site education in communal dwellings and construction sites, 2) Bear Aware educational campaign in residential neighborhoods, and 3) elevated law enforcement at two levels in the core business area of Aspen. We measured human behaviors as the response including: violation of local wildlife ordinances, garbage availability to bears, and change in use of bear-resistance refuse containers. As implemented, we found little support for education, or enforcement in the form of daily patrolling in changing human behavior, but found more support for proactive enforcement, i.e., dispensing warning notices. More broadly we demonstrated the value of gathering evidence before and after implementing conservation actions, and the dangers of measuring responses in the absence of ecological knowledge. We recommend development of more effective educational methods, application of proactive enforcement, and continued evaluation of tools by directly measuring change in human behavior. We provide empirical evidence adding to the conservation managers' toolbox, informing policy makers, and promoting solutions to human-wildlife conflicts.

## Introduction

In recent years, several authors called upon the conservation community to apply evidence-based conservation in order to maximize the use of limited resources, direct policy, and advance the field of conservation biology [1]–[3]. This call for evidence-based decision-making continues to resonate and was reiterated recently in the May 2010 issue of *Frontiers in Ecology and the Environment* [4]. This is especially true for the growing discipline of human-wildlife conflict [5]–[8], where, despite the potentially grave implications to public safety and wildlife populations, management is sometimes administered based on personal experience and expert opinion rather than evaluation. If substantial resources are expended with little impact, then conservation practitioners risk a decrease in agency credibility, an increase in public frustration, and ultimately hindrance to long-term solutions to human-wildlife conflicts [9].

Conservation tools to resolve human-wildlife conflicts are traditionally targeted at wildlife (e.g., removal, translocation, and aversive conditioning), but often have limited, short-term success [10]–[13] and lack social tolerance with stakeholders [6], [9], [14]–[15]. Therefore, there is a growing recognition among conservation biologists and wildlife managers that long-term solutions should include altering human behaviors [16]–[17]. Fall and Jackson ([6], p.89) captured this sentiment stating that “Most ‘new’ animal problems. are ones that human create and could solve by modifying their own behavior…” Public education and enforcement of wildlife-related laws are two primary methods for changing human behaviors, and despite common implementation, little research has been conducted to evaluate whether these strategies are achieving their intended goal of altering behaviors [18]–[19].

Education is considered the panacea for conflict resolution and is frequently recommended as a management tool (e.g., [7], [16], [20]). Research evaluating education has focused primarily on changing attitudes, behavioral intents, and knowledge towards wildlife and conflicts (e.g., [18], [21]). Unfortunately, there is not always a direct link between attitudes, intents, and knowledge and actual change in behavior [22]. Furthermore, studies evaluating the efficacy of education often rely on self-reported data collected via surveys [17]. As such, these studies can include a self-reporting bias [23] and lack a direct measure of human behavioral change [17]. Wildlife ordinances and laws are commonly passed to alter human behavior and reduce human-wildlife conflict, and are generally viewed as an important tool in wildlife management and conflict resolution [19], [24]. Studies evaluating the efficacy of enforcement have focused to date on enforcement of overfishing in Europe and North America, or of illegal poaching of wildlife in Africa [19], [25]–[26]. In these studies researchers compared enforcement effort to rates of illegal take, but rarely utilized an experimental approach.

In this study we experimentally evaluated public education and law enforcement in a system where humans and black bears (*Ursus americanus*) coexist but commonly come into conflict, and where wildlife agencies and municipalities employ both strategies to directly alter human behavior and reduce human-bear conflicts. Human-bear conflicts are increasing worldwide for many ursids [27]–[29], and considering that wildlife agencies prefer to target management at humans rather than bears [16], the gap in knowledge about the effectiveness of education and enforcement is especially glaring for management of human-bear conflicts. We thus collaborated with the local wildlife agency, municipalities, businesses, and residents, to experimentally test the efficacy of on-site education, a neighborhood-wide Bear Aware education campaign, and two levels of elevated law enforcement in changing human behavior.

## Methods

### Study Site

**Figure 1:**
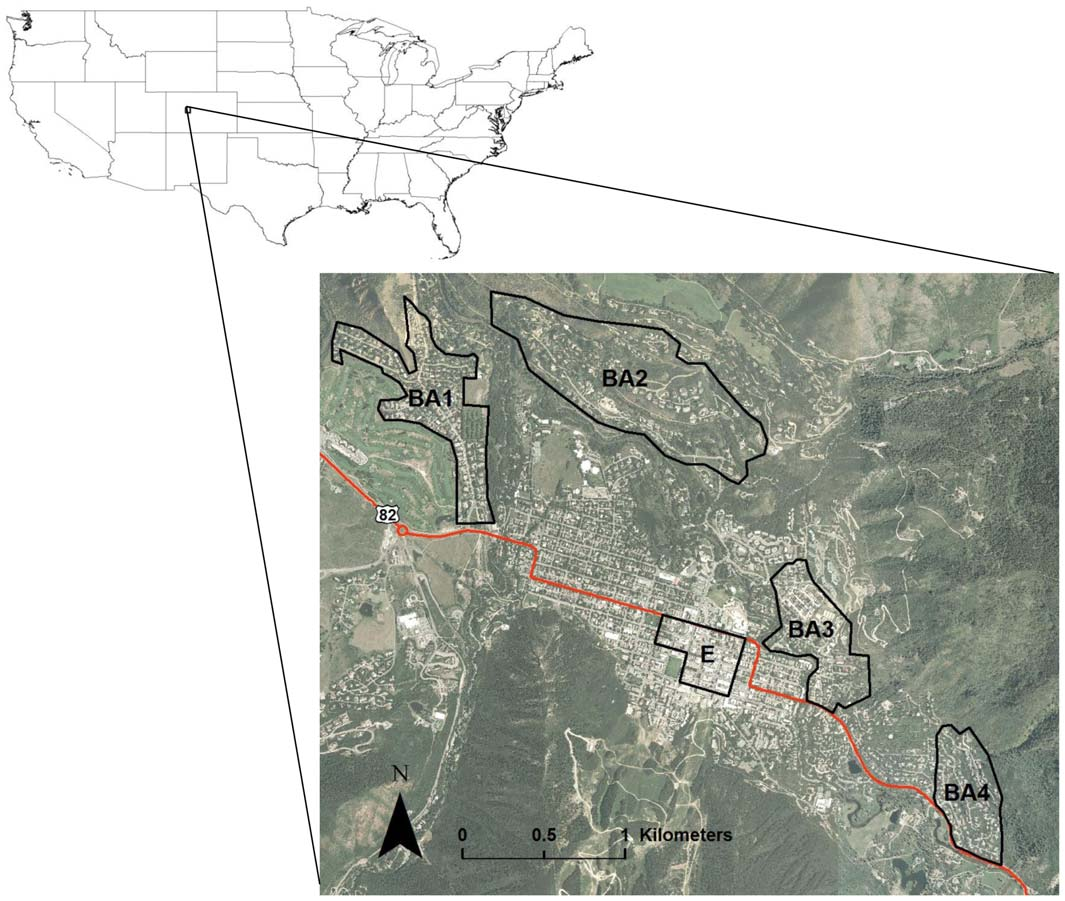
Study area. Aerial image [51] of the city of Aspen, Colorado, USA and its surrounding residential areas where experiments were conducted in 2007 and 2008 to evaluate efficacy of education and law enforcement in reducing availability of garbage to bears. Polygons represent sampling areas for the Bear Aware (BA) and Enforcement (E) experiments, where BA1-4 respectively correspond to Cemetery Lane, lower Red Mountain, lower Smuggler, and Mountain Valley neighborhoods, and E is the core business area.

We conducted experiments in the city of Aspen and surrounding residential areas of Pitkin County, located in the central Rocky Mountains of Colorado, USA (hereafter collectively referred to as Aspen; Figure 1). Aspen is situated at the confluence of four major riparian areas, Maroon, Castle, and Hunter Creeks and the Roaring Fork River, at an elevation ranging from 2,300–3,150 m. Vegetation communities include aspen (*Populus tremuloides*), lodgepole (*Pinus contorta*), Douglas fir (*Pseudotsuga menziesii*), and spruce (*Picea* spp.)-subalpine fir (*Abies lasiocarpa*) forests with pure and mixed patches of Gambel oak (*Quercus gambelli*), serviceberry (*Amelancier alnifolia*), and chokecherry (*Prunus virginiana*) shrub communities.

The Aspen town core consists of a business district and dense residential areas that gradually change into dispersed residential neighborhoods (Figure 1), and the 2007 resident population was 6,403 [30]. One third of the 4,354 total housing units in Aspen were either vacant or used for seasonal, recreational, or other uses [31], and monthly occupancy of residences was highest in July and August based on a 21-year average [32]. Fifty-one percent of the population worked in service, sales, and construction industries, and 61% commuted to work via car or public transportation [33]. The city had extensive year-round tourism with >8,800 visitors in 2008 who stayed an average of 5.8 nights [32]. In summary, the human population residing and working in Aspen was temporally dynamic and included first- and second-homeowners, seasonal workers, tourists, and service and construction industry workers that traveled daily to and from town.

Most conflicts between humans and bears in Aspen result from bears feeding on human refuse (S. Baruch-Mordo, unpublished data). Therefore, the city of Aspen and Pitkin County passed ordinances in 1999 and 2001, respectively, mandating the proper storage of any wildlife attractants including trash (City of Aspen Title 12 Solid Waste - Chapter 12.08 Wildlife Protection; Pitkin County Title 6 Health and Safety - Chapter 6.44 Wildlife Protection). Violations of the ordinances were punishable by a fine of up to US $1,000 and/or imprisonment of up to a year. With the exception of residential curbside pickup, the city ordinance required waste to be properly disposed and secured at all times in wildlife-resistant refuse collectors. The city forbade overnight placement of residential trash, and allowed curbside placement only between 0600 and 1800 hours on the day of garbage collection. The county ordinance required waste to be properly disposed and secured in wildlife-proof refuse collectors at all times, and starting in 2007, this included residential, curbside trash. Wildlife-proof containers were always required by the county for human food waste considered edible by wildlife at construction sites, but the city also allowed storage in other containers if emptied at the end of each workday.

### Refuse collectors

We define *dumpsters* as large, stationary refuse collectors constructed from metal materials that were either free standing or placed in semi-open or closed enclosures. We define *containers* as smaller capacity, movable refuse collectors mostly constructed from rigid plastic material that were used for curbside pickup.

**Figure 2:**
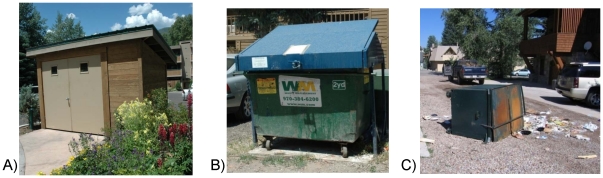
Examples of refuse collector designs. Designs that are considered bear-proof in the city of Aspen and the surrounding residential area of Pitkin County including: A) A low-risk dumpster room with metal doors, round handle, and little door clearance, B) a medium-risk dumpster with bolted, metal lid over a free-standing dumpster, and C) a high-risk free-standing dumpster with top-bar securing method that was toppled and broken into by a bear.

For dumpsters, different storage designs resulted in differential risk of break-in by bears, and we qualitatively assessed a dumpster's break-in risk using low, medium, and high criteria (Figure 2). Low-risk designs were considered most bear-proof and included garbage compactors or enclosures with features such as non-chewable metal doors, airtight construction, and round doorknobs. Medium-risk designs included bolted, encasing metal lids over a freestanding dumpster, or enclosures with wooden doors and closing mechanisms other than a round handle (e.g., locking bars or latches). High-risk designs included freestanding dumpsters or semi-open enclosures allowing bears maximum ability for manipulation and break-in; this also included dumpsters with non user-friendly securing methods (e.g., heavy lids) that led people to frequently leave them unsecured.

### On-site education experiment

We conducted the on-site education experiment at communal housing complexes and construction sites. We sampled 68 communal housing complexes, with half (34) randomly selected as treatment, and 42 construction sites with 22 randomly selected as the treatment and 20 as the control (for a detailed description of sample size determination see Appendix S1). We applied educational signs in English and Spanish on all approachable sides of treatment dumpsters. Signs had two Colorado Division of Wildlife (CDOW) messages “Garbage kills bears – Stash your trash!”, and “Help keep wildlife wild by securing the trash receptacle properly”, where we adjusted the former message for construction dumpsters as “Garbage kills bears – No food items!” Signs included colored photos of bears climbing in and out of dumpsters to illustrate their capacity to pursue trash, and a photo of a sow and cub for emotional appeal. We added a website link, that was designed for the experiment and contained information about wildlife ordinances in English and Spanish, what to do regarding violations, and Bear Aware information. We detected only one visit and only to the English version site throughout the experiment.

We sampled dumpsters July-September 2007 for three weeks each in pre- and post-treatment periods. We randomly selected four sampling days during each week for a total of 24 sampling occasions. On each visit we recorded whether the dumpster was in violation (1) or compliance (0) with the ordinances, and whether it was empty or not. A violation at a construction site entailed observing any human food trash in the open construction dumpsters. A violation at a communal housing site consisted of presence of trash items just outside, on top, or near the dumpster, or observing a dumpster that was open, or otherwise improperly secured. Because of the many refuse collector designs (see Refuse Collectors section), we additionally categorized the degree of violation at communal housing dumpsters as low, medium, and high, with low indicating little chance for a bear break-in, medium indicating with some work a bear could break-in, and high indicating a bear could easily obtain the garbage. We implemented a conservative approach where a communal housing dumpster was considered compliant if it was rated as low violation or if it was empty due to assumed recent trash collection; otherwise, dumpsters were considered in violation.

We collected covariate data on: 1) the presence of other signs about proper garbage storage, 2) the number of bear visits to a dumpster based on fresh evidence, and 3) the number of visits by Aspen Police Department (APD) or Pitkin county authorities due to bear incidents. We predicted a reduced treatment effect at sites with a previous educational sign and an enhanced treatment effect at sites with a bear incident and subsequent response by authorities. For communal housing, covariates also included the qualitative dumpster break-in risk and the number of units in the complex. We predicted greater probability of violation at higher-risk dumpsters and at complexes with more units. For construction sites, we included whether or not the site had a separate bear-resistant or bear-proof container for human food waste, and we predicted a greater treatment effect at construction sites without such a container. Finally, as part of a separate study, a survey about attitudes of residents towards bears and preference for management actions was conducted during our experiment at some apartment complexes [34]. Hence we also incorporated the number of units visited during the survey as a covariate to account for potential negative bias in violations due to increased awareness of human-bear conflicts.

We modeled the probability of violation in communal housing complex and construction site dumpsters as a function of fixed treatment and covariate effects, and a random site effect (PROC GLIMMIX) [35]. We examined correlation of covariates using Variance Inflation Factors (VIF), and used VIF >10 to eliminate correlated covariates [36]. We estimated model fit with an *r^2^* equivalent method [37], as one minus the ratio of sum of squares between the global and intercept-only models. We determined support for an effect by examining whether the 95% confidence interval of each parameter estimate overlapped zero, and we report fixed effect F-test statistics.

### Bear Aware education

We conducted the Bear Aware experiment in four residential areas: Cemetery Lane (BA1), lower Red Mountain (BA2), lower Smuggler Mountain (BA3), and Mountain Valley (BA4; Figure 1). We randomly assigned addresses in the BA1 and BA2 neighborhoods to receive the treatment of a Bear Aware campaign, a strategy commonly employed in the USA in which volunteers visit residents to distribute educational material and talk about ways to reduce attractants and conflict. Educational material was developed by the CDOW, including door hangers, magnets, “living with bears” brochures, and a checklist about how to prevent conflicts. All material instructed residents about properly securing trash in bear-proof containers. Volunteers were trained by the local district wildlife manager using CDOW protocols, and were asked to avoid disclosing the experiment and to record the date of visit and whether education material was left or the residents contacted.

Pre- and post-sampling occurred July-September 2008 for a total of 11 sampling weeks, and we monitored >650 residences (Appendix S1). As a result of the continuous treatment application, i.e., volunteers took two weeks to canvass the treatment neighborhoods, pre- and post-treatment periods varied for each residence, lasting 3–5 and 6–8 weeks, respectively. For residences in control neighborhoods, we randomly assigned a date within the two-week treatment application period to define the pre- and post-treatment periods. Because city ordinance did not require residents to place trash for curbside pickup in bear-resistant containers, we focused measurement of the response variables on whether a container was bear-resistant and whether it was secured such that trash was available to bears (1) or not (0). If a residence had a combination of bear-resistant and non bear-resistant containers, we considered it as having a bear-resistant container with trash available to bears, unless the non bear-resistant container clearly contained only yard or recycling waste.

Monitoring residential trash containers introduced several challenges. First, because of large daily variability in container placement for curbside pickup, we determined the day(s) in the week that most garbage collection occurred in each neighborhood. For the duration of the experiment, we then sampled on these days and attempted to increase container detection by sampling early before the trash collection truck arrived. Second, sometimes ambiguity existed about the ownership of a container at an address. When this occurred, we eliminated the residence from the sample. Finally, we tried to minimize being detected and the potential to bias behavior if residents learned about our study. Consequently, we used unmarked vehicles while sampling, avoided small, narrow streets where we could easily be detected, and did not sample if people were close enough to the trash container to potentially engage in a conversation.

We analyzed data at neighborhood and residence scales. For the neighborhood analysis (*n* = 4) we summarized the probability of trash being available to bears and the proportion of bear-resistant containers for each sampling occasion. We weighted both responses by the proportion of containers detected in each sampling occasion, and assessed treatment effects by the degree of overlap between group means and 95% CI. For the residence-level analysis, we conducted two analyses, one for each measured response: 1) whether a container changed from a non bear-resistant to bear-resistant (binary variable), and 2) whether probability of garbage availability decreased (continuous variable). We used logistic regression (PROC LOGISTIC) [35] to test whether non bear-resistant containers were replaced, or not, by a bear-resistant container (sample sizes: 25, 7, 18, and 8 for BA1, BA2, BA3, and BA4, respectively; Appendix S1). For the second response variable measured, we used mixed effects modeling (PROC GLIMMIX) to assess treatment effect on the probability of trash being available to bears, where residence (site) was modeled as a random effect (sample sizes: 54, 44, 48, and 46 for BA1, BA2, BA3, and BA4, respectively; Appendix S1). We used volunteer action (i.e., volunteers made personal contact with residents or left educational material) as a covariate in both analyses and predicted a greater treatment effect for sites in which volunteers had personal contact with the residents. We assessed model performance as described above.

### Elevated enforcement

We focused our enforcement experiment in four alleyways in the business area of Aspen (E in Figure 1), which consisted of restaurants, shops, offices, and communal housing complexes that were not included in previous experiments. We randomly allocated the treatment to two alleyways (37 dumpsters) with the other two used as control (30 dumpsters). Because it was not feasible to stop all enforcement in the control areas, we considered as a control the status-quo enforcement, and as a treatment the elevated enforcement of daily patrolling by the APD with the application of further measures upon detection of violation. However, after an initial treatment period in which almost no written notices were dispensed, the APD agreed to a second treatment period in which notices were dispensed at least once a week to dumpsters in violation. Hence, there were two treatment levels – additional daily patrolling (hereafter patrolling treatment), and patrolling with notice application (hereafter notices treatment). The notice of violation was taped to the violating dumpster and required a violator's response to “discuss measures that will bring you into compliance.”

We censused dumpsters in the core area for eight weeks from 1 July – 25 August, 2008, where pre-, patrolling-, and notices-treatment periods respectively lasted for three, three, and two weeks. The likelihood of dumpsters being improperly secured increased during the day due to frequent use by downtown businesses; therefore we sampled dumpsters between 0500 and 0600 hours when no, or minimal, activity occurred. Similar to the on-site education experiment, we recorded whether dumpsters were in violation or compliance with city ordinance, and whether they were empty or not. We used the guidelines described to assess the qualitative degree of violation based on dumpster type and securing methods, where a dumpster was considered in violation if it had high or medium violation and was not empty. We grouped dumpsters assigned to treatment alleyways as treatment, and used mixed effects models to test for a treatment effect (PROC GLIMMIX; dumpster as a random effect). We also conducted a *post-hoc* analysis comparing compliance of dumpsters that received written notices (*n* = 18) and those that did not (*n* = 49) regardless of alleyway location. Finally, we qualitatively assessed dumpster risk categories (Refuse Collectors section) and summarized the number of notices given to each.

## Results

### On-site education

**Figure 3:**
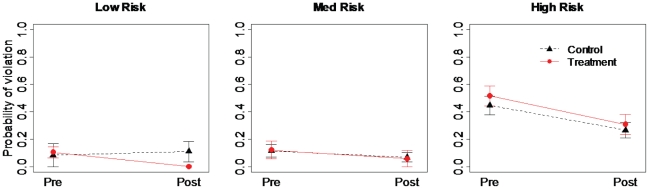
Results of on-site education experiment for communal housing complexes. Differences in mean (±1 SE) probability of violation between pre- (Pre), and post-treatment (Post) periods for dumpsters of low, medium, and high risk to break-in by bears for a 2007 experiment testing the efficacy of an on-site education sign as a management tool in reducing availability of garbage to bears in Aspen, Colorado, USA. Note: probability of violation was zero for all treatment low risk dumpsters in the post-treatment period; hence no error bars.

For the communal housing analyses we used all covariates (i.e., no correlations were detected), except number of APD visits, which was eliminated due to sparseness of data (*n* = 4). Overall, the model explained 44% of the variability in the data. The probability of a violation showed little support for a treatment effect 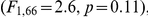 but showed stronger support for a temporal effect with a decrease in probability of violation for the post-treatment period 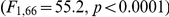 (Figure 3). A previously posted educational sign (*n_control_* = 12, *n_treatment_* = 17, ∼60% of complexes) had a slight effect on probability of violation, while bear visits to dumpsters (23 detected at 20 dumpsters) had no effect 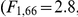 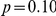 and 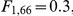 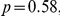
respectively). Ninety-five percent of dumpsters sampled were categorized as high (*n_control_* = 19, *n_treatment_* = 20) or medium (*n_control_* = 12, *n_treatment_* = 10) risk and high-risk dumpsters had more violations 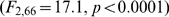 (Figure 3). The number of units in a complex 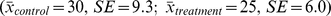 and the number of units visited by survey researchers 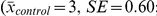 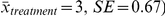 had no effect 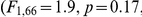 and 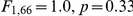, respectively).

For construction sites, projects at seven sites terminated before the end of the sampling periods and three control sites had missing covariate information, resulting in 21 treatment and 11 control sites. Additionally, no actions by the APD were recorded in the sampled construction sites, and when a bear visit was detected, we had difficulty determining the date; thus both covariates were eliminated. Overall, the model explained 28% of the variability. The probability of a violation was not influenced by the treatment 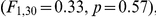 but declined (88 to 82% for control and 85 to 75% for treatment) between the pre- and post-treatment periods 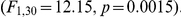 Sixteen percent of the dumpsters had a previously posted sign (*n_control_* = 2, *n_treatment_* = 3) with no effect on probability of violation 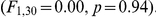 Eighteen percent (*n_control_* = 2, *n_treatment_* = 4) of dumpsters had a container for human food waste, with little effect on probability of violation 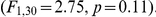


### Bear Aware education

**Figure 4:**
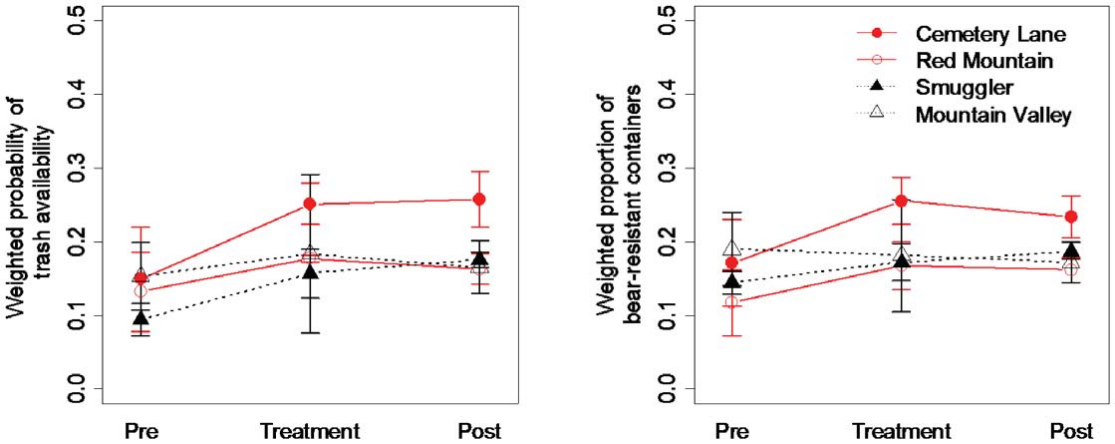
Results of Bear Aware education experiment. Differences in mean (±1 SE) weighted probability of availability of trash to bears and mean weighted proportion of bear resistant containers in treatment (red) and control (black) neighborhoods for a 2008 experiment testing the efficacy of a Bear Aware education campaign as a management tool in reducing availability of garbage to bears in four residential neighborhoods in Aspen, Colorado, USA. Responses are weighted by the proportion of containers detected in each sampling occasion. Sampling periods are pre-treatment (Pre), treatment-application (Treatment), and post-treatment (Post). Neighborhoods are Cemetery Lane (BA1), Red Mountain (BA2), Lower Smuggler (BA3), and Mountain Valley (BA4).

Volunteers spent two weeks in treatment neighborhoods visiting 235 (91% of residences) and 122 (87% of residences) addresses in the BA1 and BA2 subdivisions, respectively, while directly contacting 36% and 25% of the residences. We detected no difference in the probability of availability of trash to bears or the proportion of bear-resistant containers between control and treatment groups (Figure 4). The percent (19) of non bear-resistant containers that changed to bear-resistant ones was the same as the percent of bear-resistant containers that changed to non bear-resistant ones. For the residence analyses, the Bear Aware treatment had no effect on residents changing non bear-resistant containers to bear-resistant ones (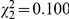, *p* = 0.95), however the model explained <1% of the variability in the data. In addition, the treatment did not reduce the probability of trash being available to bears 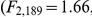 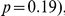 with the model explaining 10% of the variability in the data.

### Elevated enforcement

**Figure 5:**
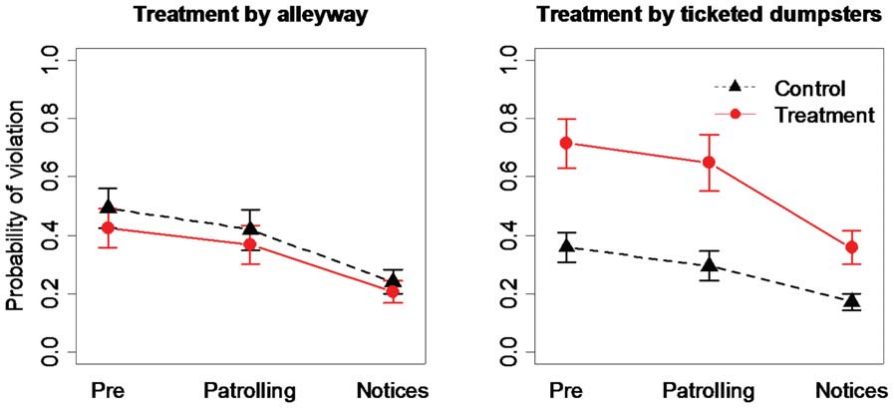
Results of elevated enforcement experiment. Differences in mean (±1 SE) probability of violation in core area dumpsters by treatment group for the pre-treatment (Pre), daily patrolling treatment (Patrolling), and written notices treatment (Notices) periods for a 2008 experiment testing the efficacy of enforcement as a management tool in reducing availability of garbage to bears in the core business area of Aspen, Colorado, USA. Treatment by alleyway included all dumpsters in the daily patrolled alleyways as treatment, whereas treatment by ticketed dumpsters included only dumpsters receiving written notices as treatment.

The APD gave 22 written and 2 verbal warnings in the treatment area, of which 4 and 20 were given during the patrolling- and notices-treatment periods, respectively. In addition, one dumpster in the control area inadvertently received three written warnings. Most dumpsters (78%) receiving tickets were high risk, and a written warning resulted in approximately 40% reduction in the probability of violation for the ticketed dumpsters between pre- and notices-treatment periods (Figure 5). We found no evidence for a treatment effect when grouping and modeling all dumpsters in the treatment alleyways as the treatment group 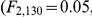 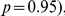 but did detect a period effect 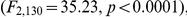 with the model explaining 30% of the variability. We found greater support for a treatment effect when grouping and modeling dumpsters receiving a written notice as the treatment 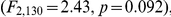 with the model explaining 31% of the variability (Figure 5).

## Discussion

Evidence-based conservation is critical to assess effectiveness of management, guide policy, and help resolve conflicts. In this study, we experimentally evaluated education and law enforcement management tools commonly used to change human behavior to reduce human-wildlife conflicts. We found that as currently implemented in our system, education had little impact in changing human behavior, while proactive enforcement was more effective in altering human behavior. We also found that it is paramount to include a rigorous monitoring protocol in order to adequately evaluate management actions.

Whether applied at specific sites, or in broad campaigns, education is often the preferred management tool to reduce conflicts between humans and wildlife (e.g., [16], [20]), and although costs can be substantial, there has been little evidence to its effectiveness. Our findings are similar to results reported from New York, where a Bear Aware education campaign had no effect in changing human behavior in better securing bear attractants [18]. Other studies focused on education showed mixed results (e.g., [21], [38]), but no studies focused on human-wildlife conflicts related to human development or explicitly measured change in human behavior. One potential explanation for our result is that the message and delivery were not adequate. For example, the low use of our education website during the on-site experiment was perhaps due to the need to write down the website address at the dumpster location. However, the education message in our signs was less likely the cause, because it included basic elements of information delivery including factual, emotional, moral, and nonverbal elements [39]. Additionally, in delivering our educational information in Spanish, we ensured that our message could reach the diverse Aspen population [40]. Regardless, the methods of education that we applied are commonly used by conservation managers, suggesting that current management methods are not effective.

Conservation biologists and wildlife managers should therefore develop new education approaches to better deliver the information. Both new and existing methods need to be continually evaluated for delivery and content, ideally by incorporating social science studies to evaluate material reception and retention [17]. Education could also be coupled with enforcement to increase its effectiveness in changing human behavior. Studies in game theory review the strategies of reward and punishment in achieving collaboration between unrelated individuals [41]–[43], and can guide the development of programs aimed to improve public cooperation. Examples for the implementation of joint education and enforcement programs include campaigns aimed at reducing underage smoking [44], increasing seatbelt wearing [45], and decreasing the use of alcohol while driving [46].

In past years, citizen-based groups and wildlife agencies have promoted the passage of wildlife ordinances as a means of reducing human-bear conflicts. An implicit assumption with respect to the passage of natural resources laws, ordinances, and regulations is that they will bring about compliance without active enforcement [19], [47]. Our study and others suggest the contrary. For example, legal protection alone had no effect on whether hunters poached protected wildlife in Africa [26], and passage of wildlife ordinances alone failed to reduce the availability of attractants, and therefore human-bear conflicts, in several North American communities [24]. Theory related to enforcement examined strategies related to increasing detection of violations (e.g., increasing patrolling efforts) and increasing penalties (e.g., increasing fine amounts) in successfully promoting compliance [19], [42]. Researchers found that increasing detection of violations, followed with proper enforcement actions, will best improve compliance with wildlife protection laws [19], [47]. Additionally, an inverse relationship was noted between the amount of enforcement resources expended to detect violations (e.g., budget spent and patrolling time) and wildlife poaching in Africa [25]–[26]. We evaluated two enforcement levels, one consisting only of elevated patrolling, and one in which written notices provided an indication of the detection of a violation by enforcement authorities. The latter brought about better compliance, suggesting that proactive enforcement in the form of notice application is necessary.

Increased patrolling, detection, and application of warnings can be costly to implement [19]. However, the alternative costs of continuously managing human-wildlife conflicts are also substantial, e.g., personnel costs, damage costs, indirect costs to human health and safety, and potential costs to the wildlife resource. The CDOW spent >5,000 hours and US $200,000 responding to human-bear conflicts in the Aspen region in 2009 alone, and the International Association of Fish and Wildlife Agencies [48] reported that wildlife agencies increased expenditure to manage human-bear conflicts by 45%, including a 22% increase in personnel time. Urban residents also incur substantial costs with damages from wildlife conflicts amounting in the USA to approximately 4 billion USD in 1993 [49]. In a review of management strategies implemented to reduce human-bear conflicts in several municipalities, Peine [24] summarized that the impetus for conflict management policy formulation and enforcement often followed a specific injury event or economic and public health concerns. Therefore, addressing violations via proactive enforcement could reduce long-term management costs and prevent additional risks to human health and safety.

We stress two important considerations for future studies when evaluating management tools: direct measures of human behavior as a response, and application of rigorous experimental design. Because conflicts arise due to a combination of factors, it can be erroneous to equate a reduction in conflicts with success of management actions without a direct measure of change in human behavior [17], [50]. For example, in our system a 2007 outbreak of human-bear conflicts resulted in an education campaign and the passage of emergency ordinances. Then in 2008, few conflicts were reported, leading some to argue that the reactive measures were successful in changing human behavior. However, despite the measures applied in 2007, ordinance violation rates in 2008 were high with relatively low use of bear-proof containers (Figures 4 and 5). Additionally, movements of GPS-collared bears showed usage shifted to areas outside of town and likely contributed to the decline in conflicts. Such confounding stresses the need for direct measurement of change in human behavior to evaluate conservation management tools [17]. But even when change in human behavior is directly observed, without proper experimental design causation cannot be inferred. For example, in our on-site education experiment we observed a strong reduction in probability of violation for both treatment and control groups, indicating that factors other than our treatment contributed to the decline. Without a control group, we could have erroneously concluded that the treatment was effective. In fact, the observed declines in 2007 likely resulted from increased probability of personal experience with bears, e.g., sighting or property damage, which could have resulted in increased awareness of bears and the change in human behaviors to better secure attractants. We therefore additionally stress the importance of applying an experimental approach when testing the efficacy of conservation tools.

This study provides evidence that current agency and municipality efforts are not necessarily effective in changing human behavior. We suggest that the conservation community can increase efficacy of management tools by coupling education and enforcement into new management programs based on insights from game theory research [41]–[43] and existing examples of society's efforts to change human behavior [44]–[46]. To effectively reduce human-wildlife conflicts or solve other pressing wildlife management issues, we also argue for increased evidence-based conservation efforts that evaluate and refine management tools to promote the coexistence between humans and wildlife.

## Supporting Information

Appendix S1 A detailed description of sample size determination.
(DOC)
