# Supplementary material for: The Carrot or the Stick? Evaluation of Education and Enforcement as Management Tools for Human-Wildlife Conflicts
Source: PLoS One. 2011 Jan 12;6(1):e15681. doi: 10.1371/journal.pone.0015681 (PMC3020222; doi:10.1371/journal.pone.0015681)
Supplement: Appendix S1 — A detailed description of sample size determination. (DOC) [file pone.0015681.s001.doc]

**Appendix S1.** A detailed description of sample size determination.

On-site education experiment

We focused the on-site education experiment on communal housing and construction sites based on GPS data showing high bear use of dumpsters in those sites (S. Baruch-Mordo, unpublished data). For communal housing, the sampling frame consisted of 437 complexes based on a GIS layer composed by the city of Aspen and Pitkin County. We applied the following inclusion criteria, complex: 1) was not a hotel nor contained any businesses, 2) had ≥5 units, 3) had a centralized dumpster not easily accessible to the general public, and 4) management granted permission for the experiment. This reduced our sample to 68 complexes, with half (34) randomly selected as treatment. Based on power analyses, this sample size allowed detection of 16.5% difference in means with 90% power (α = 0.05, hypothesized pre-treatment mean of 55% compliance based on K. Wright, Aspen District Wildlife Manager, personal comm.).

For construction sites, we obtained permission to sample all sites within the city limits. We censused all sites in Aspen, and then applied the following inclusion criteria, the construction: 1) project lasted the duration of the study, 2) site included a commercial type dumpster approximately 2-m high x 2.5-m wide x 10-m long and 3) dumpster was not easily accessible to the general public. This reduced our sample to 38 construction sites in the city limits. We augmented the sample with sites within the county from the 2004-2007 building permit database (*n* = 99), selecting sites based on our criteria (*n* = 30), and obtaining permission from landowners (*n* = 4). This resulted in a total of 42 sites, with 22 randomly selected as the treatment and 20 as the control, and allowed for detection of up to 17.5% difference in means (power = 0.9, α = 0.05, starting compliance = 20% K. Wright, personal comm.). After sampling initiation, seven construction sites terminated before the end of the sampling periods. In addition three control sites had missing covariate information. Therefore we excluded these seven sites from the analysis, resulting in 21 treatment and 11 control sites, and a 90% power to detect approximately 20% difference in means.

Bear Aware education

We conducted the Bear Aware experiment in four residential areas: Cemetery Lane (BA1), lower Red Mountain (BA2), lower Smuggler Mountain (BA3), and Mountain Valley (BA4; Figure 1). We delineated the neighborhoods such that they were isolated from other residential areas in their vicinity, and excluded any non-residences, a trailer park, and communal housing complexes included in the on-site education experiment conducted in the previous year. The sampling frame consisted of addresses in the city of Aspen and Pitkin county GIS layer that intersected with each neighborhood polygon for a total of 258, 140, 169, and 121 residences in BA1, BA2, BA3, and BA4, respectively.

For the residence-level analysis in which we tested whether treatment affected the change of a non bear-resistant container to a bear resistant one, we used data from residences for which we detected a non bear-resistant container in the pre-treatment period, and for which we detected a container in both pre- and post- treatment periods. Resultant samples sizes consisted of 25, 7, 18, and 8 residences for BA1, BA2, BA3, and BA4, respectively, and were sufficient to detect approximately 5-15% difference in proportion of bear-resistant containers with 80% power (α = 0.1, starting mean based on pilot data for each neighborhood ranging from 17.4 – 46.9% non bear-resistant containers). For the residence-level analysis in which we assessed treatment affect on the probability of trash being available to bears, we used data from residences with a container detected in both the pre- and post- treatment periods (sample sizes were 54, 44, 48, and 46 residences for BA1, BA2, BA3, and BA4, respectively).

Elevated enforcement

Trash in the core business area of Aspen was deposited in dumpsters located in four alleyways between the main streets. The sampling frame consisted of 67 bear-resistant refuse collectors. We randomly assigned the treatment to 37 dumpsters in two out of the four alleyways, leaving 30 dumpsters as control and allowing detection of <5% difference in probability of violation with 90% power (α = 0.05, starting mean based on pilot data = 43% violation). We conducted two analyses one grouping the 37 dumpsters assigned to treatment alleyways as treatment, and a *post-hoc* analysis comparing compliance of dumpsters that received written notices (*n* = 18) and those that did not (*n* = 49) regardless of alleyway location.
